# Influenza vaccine uptake among at-risk adults (aged 16–64 years) in the UK: a retrospective database analysis

**DOI:** 10.1186/s12889-021-11736-2

**Published:** 2021-09-24

**Authors:** Simon Oakley, Julien Bouchet, Paul Costello, James Parker

**Affiliations:** 1 Sanofi Pasteur, 410 Thames Valley Park Drive, Reading, RG6 1PT UK; 2 Sanofi Pasteur, Campus Sanofi Lyon Carteret, A2-6ème et. 14, Espace Henry Vallée, 69007 Lyon, France

**Keywords:** At-risk, influenza, vaccination coverage rate

## Abstract

**Background:**

In the UK, annual influenza vaccination is currently recommended for adults aged 16–64 years who are in a clinical at-risk group. Despite recommendations, rates of vaccine uptake in the UK have historically been low and below national and international targets. This study aims to analyse vaccine uptake among adults in clinical at-risk groups from the 2015–2016 influenza season to the present.

**Methods:**

A retrospective analysis of influenza vaccine coverage in the UK was conducted using data extracted from publicly available sources. Clinically at-risk individuals (as defined by Public Health England), including pregnant women, aged 16–64 years, were included in this study.

**Results:**

Influenza vaccination coverage rates across the UK in adults aged 16–64 years in a clinical at-risk group have been consistently low over the past 5 years, with only 48.0, 42.4, 44.1 and 52.4% of eligible patients in England, Scotland, Wales and Northern Ireland receiving their annual influenza vaccination during the 2018–2019 influenza season. Influenza vaccine coverage was lowest in patients with morbid obesity and highest in patients with diabetes in 2018–2019. Coverage rates were below current national ambitions of ≥75% in all clinical risk groups. In these clinical at-risk groups, influenza vaccine coverage decreased between 2015 and 2019, and there was considerable regional variation.

**Conclusions:**

Uptake of the influenza vaccine by adults aged 16–64 years in a clinical at-risk group was substantially below the national ambitions. As a result, many individuals in the UK remain at high risk of developing severe influenza or complications. Given that people who are vulnerable to COVID-19 are also at increased risk of complications from influenza, during the 2020–2021 season, there is a heightened need for healthcare professionals across the UK to address suboptimal vaccine uptake, particularly in at-risk patients. Healthcare professionals and policymakers should consider measures targeted at increasing access to and awareness of the clinical benefits of the influenza vaccine.

## Background

The influenza virus affects the respiratory tract by direct viral infection or by damage from the immune system response. Clinical presentation is highly variable, ranging from mild respiratory symptoms to pneumonia and acute respiratory distress syndrome [1].

Seasonal influenza remains a substantial contributor to the global clinical burden of lower respiratory tract infections and is responsible for substantial mortality, disability and economic disruption. In 2017, influenza lower respiratory tract infections were responsible for almost 9,500,000 hospitalisations and around 145,000 deaths worldwide [2]. Seasonal influenza epidemics contribute considerably to winter pressures in the UK, with severe cases resulting in hospitalisation due to complications*.* In the UK, during the 2019–2020 influenza season, low levels of influenza activity were observed in the community. However, there were 7990 excess deaths associated with influenza, and laboratory-confirmed influenza was associated with a total of 4918 hospitalisations and 1802 admissions to an intensive care unit/high dependency unit in England [3].

Influenza can affect anyone, but has the most significant impact on the health of very young children, the elderly and those with underlying health conditions (e.g., cardiovascular disease, neuromuscular disease, immunosuppressive medications and immunodeficiency) [4–7]. Pregnancy is also considered an important risk factor for severe influenza-associated illness and adverse neonatal outcomes due to several physiologic changes that occur during pregnancy [8–10]. Risk groups for influenza, therefore, include those at increased risk of exposure to influenza virus (e.g., healthcare workers [HCW]) as well as those at particular risk of developing severe disease (i.e., resulting in hospitalisation or death) [7, 11–13].

Influenza is a vaccine-preventable disease, and annual influenza vaccination is the most effective method for prevention [7, 11, 14]. Influenza vaccine efficacy changes for each influenza season due to antigenic drift and the decline in vaccine-specific antibodies, necessitating continual antigen monitoring of circulating influenza strains and annual revaccination [7, 11, 14, 15]. This is particularly relevant for individuals in clinical at-risk groups [7, 11, 12].

The objective of any influenza vaccination programme is to directly protect those who are most at risk of serious illness or death should they develop influenza, and this direct approach has demonstrated efficacy in reducing the incidence and burden of influenza in those vaccinated, including the elderly and immunocompromised populations [16–20]. Animal studies have demonstrated that influenza vaccines provide additional benefits by limiting shedding and transmission of influenza virus [21–23]. Therefore, vaccination can also reduce transmission and provide indirect protection to vulnerable populations [24]. Vaccination of school children provides direct protection for the recipients and indirect (herd) protection for the community [25–28], and vaccinating HCWs reduces influenza transmission to vulnerable patients [29–31]. Indeed, quantification of the direct and indirect effects of influenza vaccination suggest that for complications such as death due to influenza, which occur much more frequently in unvaccinated elderly populations, indirect benefits can surpass direct ones by a factor of at least 20 [32].

Individuals at high risk of complications following an influenza infection have historically been the target for seasonal influenza annual vaccination programmes in the UK, which started in the 1960s. Subsequent programme modifications added those above 65 years of age in 2000, pregnant women in 2010 and the morbidly obese (body-mass index ≥40 kg/m^2^) in 2014; groups for inclusion in the ‘at-risk’ category are reviewed annually by the Joint Committee on Vaccination and Immunisation (JCVI) [17, 33–35]. Table 1 describes the clinical at-risk groups defined in the influenza national vaccination programme, 2020–2021 [12].

**Table 1:** Clinical at-risk groups recommended to receive the annual influenza vaccination, 2020–2021 season [12]

At-risk group	
• Individuals aged from 6 months to less than 65 years of age, in a clinical risk group such as those with: • Chronic (long-term) respiratory disease, such as severe asthma, chronic obstructive pulmonary disease or bronchitis • Chronic heart disease, such as heart failure • Chronic kidney disease at stage three, four or five • Chronic liver disease • Chronic neurological disease, such as Parkinson’s disease or motor neurone disease • Learning disability • Diabetes • Splenic dysfunction or asplenia • A weakened immune system due to disease (such as HIV/AIDS) or treatment (such as cancer treatment) • Morbidly obese (defined as body-mass index ≥40 kg/m^2^) • Pregnancy	

Successful immunisation programmes result from high vaccine effectiveness and adequate uptake of vaccines. Theoretically, targeting individuals with the highest risk of disease or its complications is one of the most effective strategies since the rate of prevented cases per administered dose would be maximised [36].

The UK has had a long-standing selective influenza vaccination programme that aims to directly protect populations at higher risk of severe disease due to influenza [12]. Children are recognised to play a key role in the transmission of influenza, and several studies have modelled the potential effect of additional influenza prevention strategies, including paediatric vaccination programmes [37–39]. The broad consensus of these studies is that paediatric vaccination is effective. In 2012, the JCVI recommended extending the annual influenza vaccination programme to school-aged children to eventually include all children aged 2–17 years [39]. An indirect assessment suggests that since its introduction in 2013, the annual paediatric influenza national vaccination programme (NVP) has had a positive direct and indirect impact on influenza-related outcomes [28]. This supports the concept that paediatric influenza vaccination will reduce influenza-related disease in other age groups, including elderly people and individuals in high-risk groups (indirect programme impact and herd immunity).

Vaccine uptake targets are put in place to ensure sufficient direct and indirect protection within the population. They are amended regularly to ensure the protection of members of society who are unable to be vaccinated or are most likely to suffer influenza-associated morbidity and mortality.

The World Health Organization (WHO) has recently restated its aim for the highest possible level of influenza prevention and control with a focus on improving global vaccine coverage rates (VCRs) [40]. A target influenza VCR ≥75% for populations at risk of complications or those likely to transmit influenza to at-risk populations (health and social care workers) is recommended by both the WHO and the European Council [40–42]. However, many countries fail to achieve the target VCR [43].

Considered as having one of the world’s most successful influenza immunisation programmes, the VCR targets for at-risk populations in the UK is continually evolving [43]. Since 2000, VCR data have been compiled to inform evidence-based recommendations for maintaining a high VCR and improving uptake when required [43]. In light of the co-circulation of influenza and COVID-19 during the 2020–2021 influenza season, the most recent update has been to increase the UK national ambition for influenza VCR to ≥75% in all eligible populations, including clinical at-risk groups, as recommended by the WHO (Table 2) [12].

**Table 2:** Public Health England vaccination ambitions [12, 44–49]

Eligible groups	National uptake ambitions		
	2018–2019	2019–2020	2020–2021
≥65 years	≥75%	≥75%	≥75%
At-risk <65 years	≥55%	≥55%	≥75%
Pregnant	≥55%	≥55%	≥75%
Children aged 2–3 years old	≥48%	≥50%	≥75%
All primary school-aged children and school year 7 in secondary school	≥65%	≥65%	≥75%
Frontline health and social care workers	≥75%	≥80%	100%

Because the impact and the relative risk of hospitalisation attributable to influenza are substantially higher for adults in clinical at-risk groups [4], it is paramount to maintain high influenza vaccine uptake in these groups. Data from our initial review of influenza VCRs in clinical at-risk groups in the UK during the 2017–2018 season provide a snapshot and suggest that uptake is suboptimal across all clinical at-risk categories [50]. This current retrospective database analysis aims to further define influenza VCRs among clinical at-risk adults aged 16–64 years across the devolved nations of the UK from the 2015–2016 influenza season to the most recent season for which data are available (2019–2020 in England and 2018–2019 in Scotland, Wales and Northern Ireland). We further aim to identify any regional variations and variations between different clinical at-risk groups.

## Methods

The primary objective of this retrospective study was to examine annual influenza VCRs among high-risk adults aged 16–64 years in clinical at-risk groups as defined by Public Health England over five influenza seasons (2015–2016 to 2019–2020). Data were extracted from the following publicly available sources in the UK: Public Health England, Public Health Scotland, NHS Wales and Health and Social Care Northern Ireland. Data were available from all sources for the 2015–2016, 2016–2017, 2017–2018 and 2018–2019 seasons. In addition, data were available from Public Health England and Public Health Scotland for the 2019–2020 season.

We reviewed annual reports of vaccine uptake as published by Public Health England, Public Health Scotland, NHS Wales and Health and Social Care Northern Ireland. We then extracted aggregated information on vaccine uptake for patients aged 16–64 years in clinical at-risk groups eligible for vaccination. Vaccine uptake data are reported as a percentage value based on the number of eligible patients and the number of people receiving the vaccine for each eligible group.

In addition to vaccine delivery through GPs, since 2015–2016 pharmacies and other providers in the UK have also offered influenza vaccination. To capture these data, pharmacies and other healthcare providers are required to report any vaccine administration to the patient’s registered GP, and the GP will input this into the ImmForm system.

The annual data reports on the cumulative number of patients who have had at least one dose of influenza vaccine during the influenza season (1 September to the 29 February). At-risk groups are as defined in the Green Book [17] and coded via International Classification of Diseases (ICD) within the GP records.

It is important to note that the data collected for each of the devolved nations of the UK do not allow for a direct comparison, owing to slight differences in the data collection process. In England, the Public Health England (PHE) Influenza Surveillance Team collates vaccine uptake data using the ImmForm website2. In Wales, influenza vaccine data are reported by Health Board and NHS Trust Occupational Health Departments and are also collected directly from GP practices using the Audit+ software. In Northern Ireland, the flu surveillance team collects data on the number of people vaccinated in each target group and VCRs are calculated using age-specific denominators. This study describes trends in VCRs and did not involve formal statistical analysis.

## Results

At the time of analysis, data for the 2019–2020 influenza season were available for England and Scotland, while data for the 2018–2019 influenza season were available for all four of the devolved nations. Comparisons in this retrospective analysis of influenza vaccine coverage in the UK primarily focus on data obtained from the 2018–2019 influenza season. In England, PHE reports on the number of patients aged 6 months to under 65 years in a clinical risk group (excluding pregnant women without other risk factors and carers) and who received influenza vaccine. Overall, the numbers of patients aged 6 months to under 65 years in a clinical risk groups has remained relatively stable over the past five influenza seasons (Table 3).

**Table 3:** Number of patients aged 6 months to <65 years in a clinical risk group registered and who received influenza vaccine

	2015–2016	2016–2017	2017–2018	2018–2019	2019–2020
**Number of patients registered**	6,821,163	6,175,910	6,836,969	6,820,919	7,086,331
**Number of patients vaccinated**	3,079,134	3,003,867	3,344,593	3,276,592	3,182,752
**Vaccine uptake**	45.1%	48.6%	48.9%	48.0%	44.9%

### Influenza vaccine uptake rates in at-risk adults aged 16–64 years over time

**Fig. 1:**
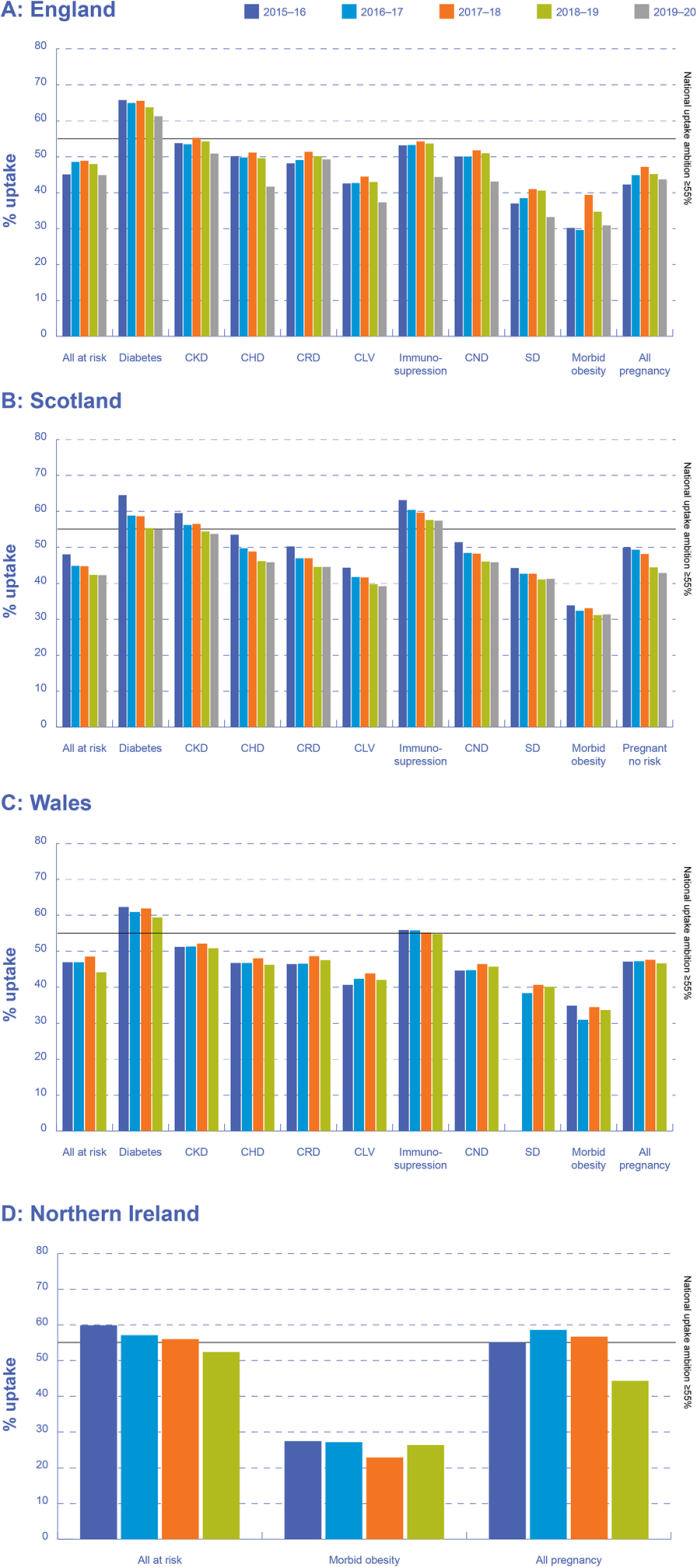
Influenza vaccine uptake rates in at-risk groups across the UK, 2015–2020. CKD, chronic kidney disease; CHD, chronic heart disease; CRD, chronic respiratory disease; CLD, chronic liver disease; CND, chronic neurological disease; SD, splenic dysfunction (including asplenia). Note: Data from Public Health Scotland, NHS Wales and Health and Social Care Northern Ireland for the 2019–2020 season were not available. Data from Public Health Scotland and Health and Social Care Northern Ireland for specific clinical risk groups were not available

Overall, influenza VCRs across the UK in all at-risk adults aged 16–64 years have been consistently low over the past 5 years, with uptake rates below the national ambition of ≥55% (Fig. 1). In 2018–2019, VCRs in all at-risk adults aged 16–64 years were 48.0% in England, 42.4% in Scotland, 44.1% in Wales and 52.4% in Northern Ireland. In Scotland, Wales and Northern Ireland, VCRs have decreased over successive seasons since 2015–2016. Although VCRs in England increased slightly between 2015 and 2016 and 2017–2018, uptake rates in subsequent seasons have decreased (Fig. 1).

### Influenza vaccine uptake rates in at-risk adults aged 16–64 years according to clinical at-risk group

Influenza VCRs are highly variable according to clinical at-risk group (Fig. 1). Across England, Scotland, Wales and Northern Ireland, influenza VCRs were lowest in patients with morbid obesity (34.7, 31.2, 33.6 and 26.3%, respectively, in 2018–2019), chronic liver disease (43.0, 39.8 and 42.0% [2018–2019]; data not available for Northern Ireland) and splenic dysfunction (40.6, 41.1 and 40.1% [2018–2019]; data not available for Northern Ireland). Influenza VCRs were generally higher in patients with diabetes than in other clinical at-risk groups in England and Wales, at 63.7 and 59.4%, respectively, for the 2018–2019 season. In Scotland, influenza VCRs were generally higher in patients with immunosuppression, at 57.6% for the 2018–2019 season. Although the 2018–2019 target of ≥55% was achieved for patients with diabetes, the VCRs reported are substantially below the 2019–2020 target of ≥75%.

As with overall rates, influenza VCRs have decreased over the past five seasons across almost all clinical at-risk groups, particularly in England and Scotland. This trend is particularly true in patients with immunosuppression in England (VCR of 53.2% in 2015–2016 compared with 44.4% in 2019–2020). In Wales, the influenza VCRs across most clinical at-risk groups have largely remained steady since 2015, and there has been little improvement in VCRs.

### Regional variations in influenza vaccine uptake rates in at-risk adults aged 16–64 years

In addition to variations between the devolved nations in influenza VCRs in at-risk adults aged 16–64 years (Fig. 1), interrogation of data sources in England revealed substantial regional variations in influenza VCRs, with consistently lower rates in some geographical regions. In 2019–2020 in England, influenza VCRs in all clinical at-risk patients ranged from 41.8% in London to 48.6% in Greater Manchester. At the Clinical Commissioning Group (CCG) level in England, only one of 191 CCGs achieved the national vaccine uptake target of ≥55% in 2019–2020, with vaccine uptake ranging from 28.7% in Hammersmith and Fulham to 58.1% in Stockport.

### Influenza vaccine uptake in pregnancy

**Fig. 2:**
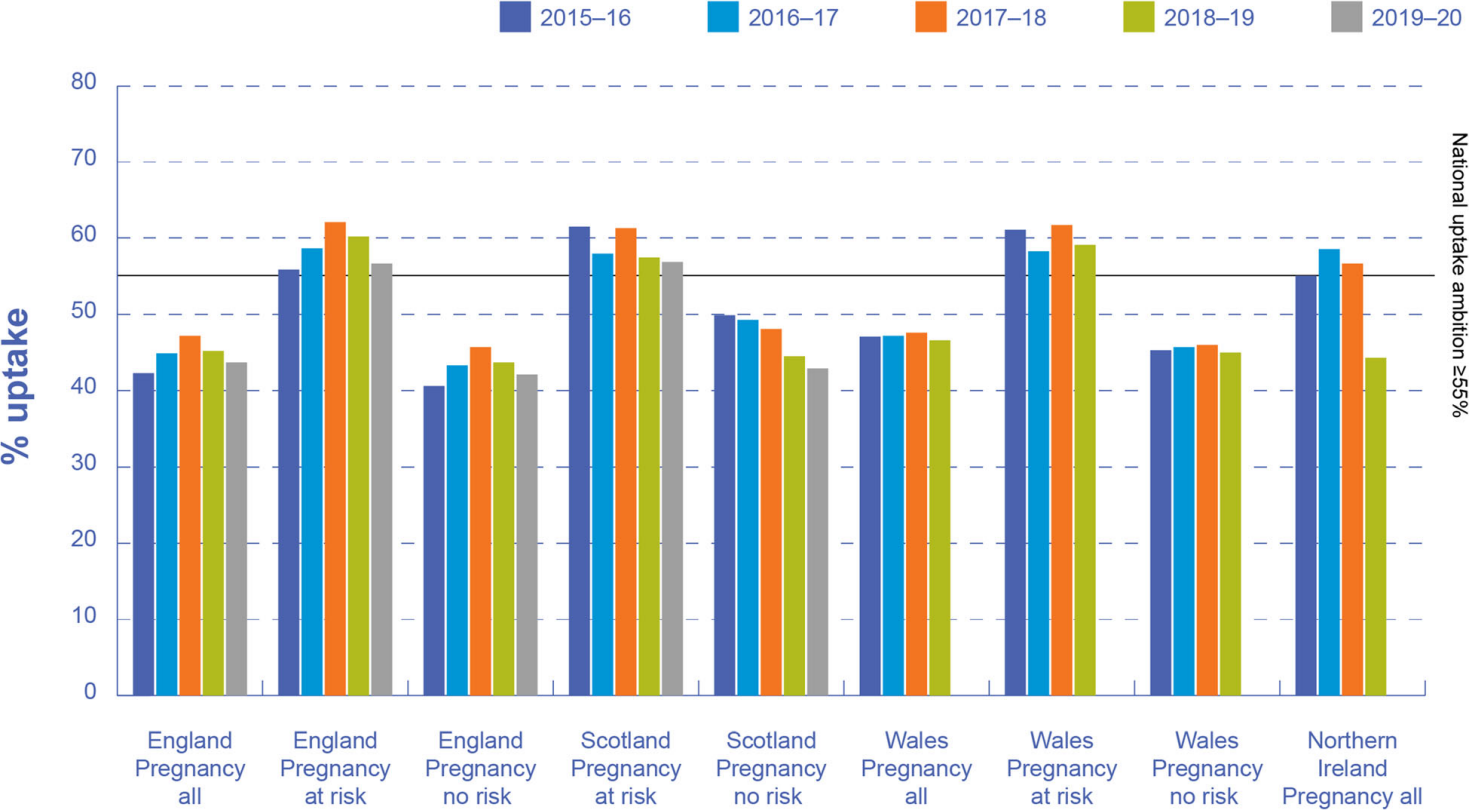
Influenza vaccine uptake rates in pregnant women across the UK, 2015–2020. Note: Data from Public Health Scotland, NHS Wales and Health and Social Care Northern Ireland for the 2019–2020 season were not available. Data from Public Health Scotland for pregnancy all were not available, and data from Health and Social Care Northern Ireland for pregnancy at risk or no risk were not available

Overall, influenza VCRs in pregnant women across the UK have been consistently low over the past 5 years, with uptake rates substantially below the pre-2020 national ambition of ≥55% in England, Scotland, Wales and Northern Ireland (Fig. 2). Despite higher influenza VCRs in pregnant women with at least one additional clinical risk factor compared with pregnant women with no additional risk factors, these remained below 60% in England, Scotland and Wales in 2018–2019 (data not available for Northern Ireland). In pregnant women with no additional risk factors, influenza vaccine uptake was 43.7, 44.5 and 45.0% in England, Scotland and Wales, respectively, in 2018–2019 (Fig. 2).

In addition to variations between the devolved nations in influenza VCRs in pregnancy (Fig. 2), interrogation of data sources in England revealed substantial regional variations in influenza VCRs, with consistently lower rates in some geographical regions. In 2019–2020 in England, influenza VCRs in pregnancy ranged from 39.2% in London to 50.2% in Greater Manchester. At CCG level in England, only four of 191 CCGs achieved the national vaccine uptake target of ≥55% in 2019–2020, with vaccine uptake ranging from 26.2% in the Isle of Wight to 68.0% in Stockport.

### Influenza vaccine uptake in England during the SARS-Cov-2 pandemic

Provisional data to week 53 (28 December to 3 January) for the ongoing 2020–2021 season suggest that seasonal influenza vaccine uptake in GP practices has increased in people aged ≥65 years (80.3% in 2020–2021 vs 67.6% in 2019–2020), at-risk patients aged 2–65 years (51.5% in 2020–2021 vs 35.5% in 2019–2020) and pregnant women (43.1% in 2020–2021 vs 37.5% in 2019–2020).

## Discussion

This analysis provides an overview of seasonal influenza VCRs in the UK between 2015 and 2020 among clinically at-risk adults aged 16–64 years. Although the VCR data for at-risk patients aged six months to under 65 years is stratified by age band by PHE and other stakeholders, this analysis aimed to describe vaccine uptake among adults (age 16 to 64 years) in clinical at-risk groups from the 2015–2016 influenza season to present. The study highlights that vaccine uptake among at-risk patients has remained stable over the past 5 years with little improvement in uptake rates over the five year period and is remains suboptimal.

Despite the national recommendations for annual influenza vaccination in clinical at-risk groups, VCRs have remained consistently below national ambitions over the past five influenza seasons. Only 48.0, 42.4, 44.1 and 52.4% of eligible patients in England, Scotland, Wales and Northern Ireland, respectively, received their annual influenza vaccination during the 2018–2019 influenza season. Similar and consistently low VCRs were also observed in pregnant women. These low VCRs are disappointing, given the increased risk of severe influenza and complications in these individuals. The analysis suggests that people in these groups would benefit from measures to increase the uptake of vaccination.

Influenza VCRs are lowest in patients with morbid obesity, chronic liver disease and splenic dysfunction. Reduced rates of influenza vaccine uptake in patients with morbid obesity have been reported elsewhere, regardless of patient age [51]. This may be explained by the relatively recent (2015) inclusion of morbid obesity as a clinically relevant risk factor in the influenza NVP and the fact that general practitioners (GPs) were not reimbursed for vaccination of patients with morbid obesity until the 2017–2018 influenza season [51].

Influenza VCRs were highest among patients with diabetes, although these rates remained substantially below the national ambition of ≥75%. We suggest this higher uptake among diabetic patients when compared with other clinical at-risk groups could be attributed to the fact that this patient group has well-defined inclusion criteria leading to better identification in GP practice registers. In addition, patients with diabetes comprise one of the largest at-risk groups in terms of patient numbers in the entire at-risk population in the UK. Therefore, this group may have been targeted for specific patient education campaigns previously. For some clinical at-risk groups, there may be a lack of patient education materials explaining the risk of severe influenza infection and complications associated with their disease.

The UK is considered a world leader in vaccine surveillance with robust reporting in clinical practice. Most influenza vaccines are delivered via primary care and uptake data are extracted automatically from GP electronic health record systems onto the relevant platforms. Data collected includes those vaccinated by another healthcare providers including community pharmacy and secondary care (provided the GP patient electronic record is updated). PHE and associated stakeholders across the devolved nations are responsible for monitoring coverage of all vaccines in the national immunisation schedule, and there are no regional differences within countries in terms of vaccine uptake data collection. Aggregated data are reported by the vaccine providers and analysed and published on a regular basis and there is a minimal time lag in reporting.

Many countries consistently fail to achieve the target influenza VCR of 75% for populations at risk of complications as recommended by the WHO and the European Council [43, 52]. Results from a survey of seasonal influenza immunisation policies (2017–2018 influenza season) in European Union (EU) and European Economic Area (EEA) Member States with a temperate climate reports that all 30 Member States recommend influenza vaccination in older people and populations with chronic medical conditions, and 28 Member States recommend influenza vaccination in pregnant women [52]. However, VCRs (2016–2017) for older populations as reported by 19 Member States (Denmark, Estonia, Finland, Germany, Hungary, Iceland, Ireland, Italy, Latvia, Lithuania, the Netherlands, Norway, Poland, Portugal, Slovakia, Slovenia, Spain, Sweden and the UK) were below WHO targets (range 2.0 to 72.8%) [52]. Similarly, VCRs for populations with chronic medical conditions as reported by seven Member States (Czech Republic, France, Ireland, the Netherlands, Norway, Portugal, and the UK) were also below WHO targets (range 15.7 to 57.1%) and VCR for pregnant women as reported in nine Member States (Belgium, Finland, Hungary, Ireland, Italy, Lithuania, Romania, Slovenia, and the UK) were below WHO targets (0.5 to 58.6%) [52].

A recent review of VCRs from four countries with influenza VCRs generally considered to be ‘high’ (Australia, Canada, UK and USA) concluded that suboptimal vaccination coverage is a complex issue that can be influenced by socio-demographic, programmatic and socio-psychological factors [43]. A total of 42 key factors for a successful influenza vaccination programme were identified and clustered into five pillars: [1] Health Authority accountability, and strengths of the influenza programme, [2] facilitated access to vaccination, [3] healthcare professional accountability and engagement, [4] awareness of the burden and severity of disease and [5] belief in influenza vaccination benefit [43].

A variety of patient-level and systems-level factors are probably associated with seasonal influenza vaccine uptake among clinical at-risk groups in this UK focussed study. Patient-level factors such as perceived susceptibility to disease and perceived vaccine effectiveness are predictors of vaccine acceptance. There is evidence to suggest that patients in high-risk clinical groups are more likely to receive an influenza vaccine after they receive information on the benefits of vaccination to their own health compared with social benefits to others. This correlation is even stronger when the patient perceives themselves as personally at higher risk [53]. In addition, system- and practice-level factors such as access to preventative care services can affect influenza VCRs in the UK. A recent study in a UK population of clinically higher risk patients highlighted access-related barriers, including timing, availability and location of appointments for vaccinations, which at least partially correlated with socioeconomic deprivation [51]. Additional system-level factors included access to GP appointments throughout the year, with higher levels of vaccine uptake in patients with higher numbers of GP appointments per year, potentially indicating the key role that GPs play in vaccine uptake and that patients who seek healthcare support are more inclined to be vaccinated [51].

A lack of understanding of the severity of influenza-related complications can impact a patient’s decision to receive the influenza vaccine; pregnant women who were not UK citizens and whose first language is not English reported communication concerns, resulting in an inability to access information on vaccination from their healthcare providers [54].

Attitudes among patients in clinical at-risk groups affect vaccine uptake; patients of all ages in at-risk groups were reportedly less likely to be vaccinated following a season with a vaccine of low effectiveness, and patients aged under 65 years were less likely to receive a vaccine following a season of high influenza severity, compared with seasons of low severity [51]. Similarly, there is evidence to suggest that the language used by healthcare professionals around herd immunity and a “one size fits all” approach to maternal vaccination contributes to a lack of knowledge, misconceptions and distrust of healthcare professionals among pregnant women from lower socioeconomic backgrounds [54].

Discrepancies in influenza vaccine uptake owing to socioeconomic status disparities have been highlighted elsewhere; higher rates of influenza-associated illness and hospitalisations, coupled with lower influenza vaccine uptake among Merseyside residents (a geographic area in the UK scoring high on Index of Multiple Deprivation taking into account housing, education, environment and crime) highlighted significantly higher influenza-related burden in people from more deprived neighbourhoods than in those from less deprived areas [55]. Similarly, data on influenza vaccine uptake between 2011 and 2016 highlighted disparities based on ethnicity, sex, age, socioeconomic deprivation and comorbidities [51].

Socioeconomic discrepancies and other factors may explain the regional variation highlighted in this study; influenza VCRs in all high-risk patients ranged from 41.8% in London to 48.6% in Greater Manchester. Regional variations were also apparent among pregnant women, with only four of 191 CCGs achieving their vaccine uptake target of ≥55% in 2019–2020. Regional discrepancies in VCRs across the UK highlight a need for improved consistency in vaccine uptake recommendations and patient education.

Every year across the UK, a sophisticated campaign is developed which aims to improve uptake rates of influenza vaccination. The strategy, developed and published as part of the annual Flu Plan, sets out a coordinated and multi-channel approach to guide the implementation of an awareness campaign and provides partner organisations with information and resources ahead of the influenza season. Several patient associations and organisations representing at-risk populations, including the British Lung Foundation, British Heart Foundation, Diabetes UK and Age UK actively support and participate in the campaign and create customised campaign resources and messages.

A 2012, nationwide, cross-sectional survey of UK general practice identified several strategies and procedures associated with higher rates of influenza vaccine uptake in at-risk patients [56]. For at-risk patients aged under 65 years, having a lead member of staff for planning the influenza campaign and producing a written report of the practice’s performance was predictive of an 8% higher influenza vaccination rate compared with practices that do not employ these strategies (54% vs 46%). The active involvement of midwives in providing influenza vaccination to pregnant women was also significantly associated with higher levels of vaccine uptake.

In 2018, the National Institute for Health and Care Excellence (NICE) published a guideline describing mechanisms to increase uptake of influenza vaccination among eligible populations, including people at high risk from influenza and its complications. These guidelines highlight the importance of a multi-component approach, raising awareness, and auditing and monitoring of vaccine uptake [57]. In 2020, NICE subsequently published a structured overview of potential quality improvement areas for increasing the uptake of the influenza vaccine [58]. Areas of improvement include addressing the scepticism around influenza vaccination, improving the delivery of advice and information (including online sources and social media), and tailoring advice and information.

Any intervention to increase vaccine uptake should be monitored and evaluated systematically, to guide development and wider implementation. Understanding the strategy or reasons behind vaccine uptake is not part of the scope of this study; the study does, however, highlight that uptake rates have not significantly changed in five years and strategies to improve uptake rates are required.

Provisional data for the ongoing 2020–2021 season suggest that seasonal influenza vaccine uptake has increased in all eligible populations, with the highest rates ever achieved in people aged ≥65 years. For at-risk patients aged 2–65 years, uptake was provisionally reported as 51.5%, a rate higher than the previous seven seasons. This data is encouraging, especially amongst groups most vulnerable to influenza and also most at risk for COVID-19 and might be a direct result of the COVID-19 pandemic. This data also supports the results of a recent observational study measuring the impact of the COVID-19 pandemic on acceptance of influenza vaccination in the 2020–2021 season. This UK-wide study, showed COVID-19 increased the acceptance of influenza vaccination in the 2020–2021 influenza season from 79.6 to 91.2% in those previously eligible [59].

Provisional data for the ongoing 2020–2021 season indicate that COVID-19 activity at a national level continued to increase while influenza activity, including GP consultations and hospital admissions, remained at or below baseline levels [60]. This is most likely due to a combination of the higher rates of influenza vaccine uptake and precautions implemented to slow down COVID-19 transmission (social distancing, hand washing and mask-wearing) that may also reduce the spread of influenza, which, similar to COVID-19, is primarily transmitted through respiratory droplets and contact with contaminated surfaces. As these precautionary measures are relaxed over time, we suggest influenza infection will increase in future seasons.

### Limitations of the study

It is important to note that the data collected for each of the devolved nations of the UK do not allow for a direct comparison, owing to slight differences in the data collection process. Data is collected from different data sources, including GP practices, schools, and Occupational Health Departments.

Other potential limitations are the exclusion of data from the prison population and healthcare and social workers if they were vaccinated outside of GP surgeries and not reported via the ImmForm system. There are were also challenges with recording patients owing to a change in GP supplier system in 2019–2020 and delays with reporting of patients vaccinated in pharmacies. Reporting of pregnant patients who receive the influenza vaccine is particularly challenging owing to delays in updating patients’ electronic records after birth or loss of pregnancy and the nature with which pregnant women enter and leave the risk group throughout the influenza season.

It is also important to note that many people will have more than one clinical risk factor that makes them eligible for influenza vaccination; for example, a patient may suffer from both diabetes and chronic heart disease. When viewing influenza vaccine uptake broken down by clinical risk group, it is important to keep in mind that the same patient may be present in a number of different risk groups; however, a patient will only be counted once in the overall total uptake figure.

## Conclusions

Uptake of the influenza vaccine by adults aged 16–64 years in a clinical at-risk group is substantially below the national ambitions, and as a result, a large number of individuals in the UK remain at a high risk of developing severe disease.

Given that people who are vulnerable to COVID-19 are also at increased risk of complications from influenza [13], during the 2020–2021 season there was a heightened need for healthcare professionals across the UK to increase vaccine uptake, particularly in clinical at-risk patients. A nationwide campaign to increase influenza VCRs in high-risk patients has the potential to save lives and reduce GP consultations, hospitalisations and pressure on accident and emergency departments.

With this in mind, healthcare professionals and policymakers should consider measures targeted at increasing access to and awareness of the clinical benefits of influenza vaccine uptake in at-risk adults, to achieve the current national vaccination ambition of ≥75% across the UK.

## Data Availability

The data supporting the conclusions in this article are available from the following publicly available data sources, as described in the methods section: Public Health England: https://www.gov.uk/government/collections/vaccine-uptake#seasonal-flu-vaccine-uptake:-figures Health Protection Scotland: https://hps.scot.nhs.uk/publications/scottish-vaccine-update/ NHS Wales: http://www.wales.nhs.uk/sites3/page.cfm?orgid=457&pid=55714 HSC Public Health Agency: https://www.publichealth.hscni.net/directorate-public-health/health-protection/seasonal-influenza

## Abbreviations

CCG: Clinical Commissioning Group
GP: general practitioner
HSCT: Health and Social Care Trusts
JCVI: Joint Committee on Vaccination and Immunisation
NVP: National vaccination programme
PHE: Public Health England
VCR: vaccination coverage rate
UK: United Kingdom
